# Mothers’ perceptions of their child’s enrollment in a randomized clinical trial: Poor understanding, vulnerability and contradictory feelings

**DOI:** 10.1186/1472-6939-14-52

**Published:** 2013-12-10

**Authors:** Adriana Assis Carvalho, Luciane Rezende Costa

**Affiliations:** 1 Health Sciences Program, Federal University of Goias, Primeira Avenida, s/n, Setor Universitario, Goiania, GO 74605-220, Brazil; 2 Faculty of Dentistry, UFG, Goiania, Brazil

**Keywords:** Dental care for children, Qualitative research, Bioethics, Randomized controlled trial

## Abstract

**Background:**

Little is known about the views of mothers when their children are invited to participate in randomized clinical trials (RCTs) investigating medicines and/or invasive procedures. Our goal was to understand mothers’ perceptions of the processes of informed consent and randomization in a RCT that divided uncooperative children into three intervention groups (physical restraint, sedation, and general anesthesia) for dental rehabilitation.

**Methods:**

This is a qualitative study based on semi-structured interviews with mothers accompanying children under 3 years old presenting severe early childhood caries. Their responses were analyzed using content analysis.

**Results:**

We identified one major theme from 15 mothers’ responses – “Understanding of, attitudes toward, and feelings about consenting to participate in a RCT involving advanced behavior guidance techniques and about randomization” – that was derived from the following subcategories: confusion in defining techniques, questions after signing the consent form, lack of knowledge about the techniques, acceptance or questioning of the drawing, sharing responsibility with the child during the drawing, and feelings of faith in God, fear, powerlessness to choose, and relief from or an increase in pressure.

**Conclusions:**

Despite mothers’ misunderstanding, vulnerability, and contradictory feelings, they were willing to overlook their thoughts in order to complete their children’s dental treatment.

## Background

Clinical trials are experiments that compare the effects of two or more healthcare interventions; they include uncontrolled trials, controlled trials and randomized controlled trials (RCTs) [1]. A RCT is carried out when there is more than one alternative treatment for a disease and it is not known which is the most appropriate [1]. The role of randomization is to eliminate selection biases and to balance the effects of confounding variables [2]. Randomization is controversial for various reasons, but particularly when a placebo is used and the investigator does not prescribe treatment on the merits of each case [2]. Randomization can be ethically justified when researchers do not know which intervention works best and an appropriate allocation can guarantee that participants are distributed equally among groups [3].

Although the number of clinical trials in pediatrics has increased, there are methodological issues regarding the conduct and reporting of the research [4]. In pediatric clinical trials, researchers should share the responsibility for beneficial or harmful treatment effects with parents [5,6]. The possibility of treatment failure arouses great anxiety and fear in parents when they are asked to decide whether or not to allow their children to participate in such research [6]. During the recruitment phase in a therapeutic trial, parents seem to place more confidence in the health professional/researcher than in the content of information packs [7].

In the pediatric dentistry field, one of the topics that warrants more RCTs is related to behavior guidance techniques. Dental treatment is associated with too many stimuli that may be perceived as harmful or uncomfortable by patients because it has the potential to negatively involve the senses: the sound of the motor hand piece, the sight of the dental setting and sharp tools, e.g., the needle, the smell of dental materials, the touch of vibrating and sometimes painful instruments, the taste of blood and rubber, and so forth [8]. Given those stimuli, a child may manifest different levels of dental treatment avoidance behavior, including verbal protests, movements of the body and head, and crying [8].

If a child lacks psychological or emotional maturity and/or has a mental, physical, or medical disability which does not allow proper cooperation with dental treatment, the dentist should use advanced techniques of behavioral guidance, which include physical restraint (protective stabilization), sedation and general anesthesia [9]. Those techniques have risks and benefits, as well as indications and contraindications, which overlap in many cases [9]. Also, as there are countries where continuing education courses on sedation and general anesthesia for pediatric dental treatment are unavailable [10], some dentists still have to physically restrain children to perform dental rehabilitation and, in consequence, eliminate the pain and improve the quality of life of the children and their families.

As the long-term effects of the advanced behavioral techniques on a child’s behavior in the dental chair are not fully understood, a multidisciplinary group of health professionals has carried out a RCT in Central Brazil to investigate the aforementioned question (registered in clinicaltrials.gov under the protocol NCT 00902395). In that study, because the premise of the trial was that there is no evidence to suggest that one procedure is preferred over another with regard to their long-term impact on children’s behavior in the dental chair, children were randomized to one of the three techniques: physical restraint, sedation or general anesthesia.

Studies have recognized that randomization may be difficult for parents because of an incompatibility between their desires and the group to which their child is allocated [6]. A few studies have focused on parents’ understanding of the clinical trial consent form [11-13]. It was determined that parents felt that the explanations and reassurances provided by dentists helped them to decide to allow their children to take part in a RCT; however, that investigation did not explore their views of the randomization method [7].

This study sought to understand the perceptions of mothers regarding the informed consent and randomization processes linked to a RCT that compared advanced behavior management techniques for pediatric dental rehabilitation. The assumption was that mothers would have difficulties in understanding the consent form and that most of them would accept the randomization because their children needed the dental treatment.

## Methods

### Study design

This study used a qualitative approach to yield exploratory, descriptive, and explanatory data regarding mothers’ feelings about the process used to allocate their children to one of three advanced behavior management technique groups. Data were collected through semi-structured interviews to explore the meanings and experiences expressed by the participants [14]. This study was approved by the Institutional Review Board (IRB) at the Federal University of Goias (UFG), Goiania-GO, Brazil, and met Declaration of Helsinki human research standards. All participants signed a specific consent form for the present study.

### Setting

This study was carried out at the Dental Sedation Center (NESO), UFG. NESO is an extension project that aims to restore the oral health of low-income children who have caries or another oral pathology but do not cooperate with dental treatment. The children, who are usually referred from the public health system, receive comprehensive dental treatment under moderate sedation (with/without physical restraint) outside the operatory room. NESO is staffed by a multidisciplinary team (pediatric dentist, dental surgeon, anesthesiologist, pediatrician, psychologist and speech therapist).

### Participants

Participants of this study were the mothers of children under 3 years old who were recruited to receive dental care under physical restraint, moderate sedation, or general anesthesia in a RCT (NCT 00902395). The children had early childhood caries, a severe oral condition that primarily causes pain and chewing difficulties and their oral problems had not been resolved by other public or private dental care services. Dental treatment with physical restraint and no sedative is considered ethical in Brazil because of the unavailability of settings to provide pharmacological behavioral guidance. Although a total of 48 children were recruited for the RCT, we limited the present study to 15 mothers because this number allowed data saturation to be reached, i.e., the answers ceased to provide additional information.

### The informed consent process for the RCT

Children and mothers were recruited to participate in the trial through notices distributed by public health services. Following the trial recruitment stage, a pediatric dentist trained to provide the consent form orally and individually explained the study to the children’s parents or guardians; the dentist then answered any questions posed by the parents and invited one parent of each child to sign the consent form if they agreed to allow their children to participate in the trial.

The consent form, which was approved by the IRB, was designed to be at an acceptable reading level for this study population; it was a two-page form that contained an explanation of the study aims, procedures, etc., including the advanced behavior management techniques that would be assigned in the randomization process (Table 1). The informed consent and randomization processes were conducted in the same day; consequently, there were no delay intervals between the obtainment of consent and the interviews.

**Table 1:** Excerpt of the informed consent form showing the explanation of the advanced behavioral management techniques

**Technique**	**Description**	**Benefits**	**Risks**
Physical restraint	Your child will be wrapped in a sheet so s/he does not move and so is not at risk of getting hurt during treatment. You can stay with him/her during the treatment, which will be performed in the dental clinic in as many sessions as needed.	No medication is used.	It can cause stress and more serious emotional problems such as dental trauma, fear or phobia.
Moderate (“conscious”) sedation	Your child will receive the sedative midazolam, administered orally 20 minutes before the procedure, which will be performed in the dental school in as many sessions as needed. In this case, an anesthesiologist will accompany the child. You can stay with him/her during treatment, and s/he will also be wrapped in a sheet to prevent any injury.	It can relieve suffering during dental treatment, cause amnesia regarding the procedure and so improve the quality of care.	It can cause nausea, vomiting, difficulty breathing, agitation in children (paradoxical reaction), dizziness, irritation in the post-operative period, allergies to the sedative, longer action of the sedative; serious respiratory and/or cardiac depression is rare.
General anesthesia	Your child will have a pre-anesthetic evaluation by an anesthesiologist and a pediatrician and will be admitted in the university hospital for one day to have all the treatment completed in one session. In this case, hospital staff will perform the anesthesia.	It can relieve suffering during dental treatment, cause amnesia regarding the procedure and so improve the quality of care.	It can be related to sore throat, hoarseness, nausea, vomiting, gastric aspiration, eye lesion, oral lesion, allergic reaction, hospital infection, changes in breathing/pressure/pulse, cardiopulmonary depression, neurological lesion, and death.

### Data collection – interviews

Interviews for the present study occurred in two phases: 1. After a parent had signed the consent form; 2. After the randomization process. Interviews took place at the NESO dental clinic.

One researcher having formal education in psychology interviewed mothers using a guide presenting open-ended questions that had been prepared in advance for each phase (Table 2); this researcher did not take part in the consent process. Mothers were interviewed individually. In two cases, there was no other accompanying adult to look after a child, and as a result, the child had to remain in the interview setting with his/her mother.

**Table 2:** Semi-structured interview guide

**Phase 1 – after the informed consent process, before the randomization**	
1.	Did you already know about these three techniques of guiding the child’s behavior during dental treatment: protective stabilization, moderate sedation and general anesthesia?
	a. If YES, please explain how you learned about them.
	b. If NO, did you understand the explanations in the informed consent process?
	c. Please explain in your own words what you understand about each of them.
2.	If you could choose one of the three techniques, what would it be? Why did you make this choice?
3.	What do you think about using the protective stabilization during your child’s dental treatment?
4.	What do you think about sedating your child for dental treatment?
5.	What do you think about your child being referred to general anesthesia to receive dental treatment?
**Phase 2 – after the randomization**	
1.	How did you feel during the drawing? And about the drawing result?
2.	How do you think your child will behave during the dental appointment considering the technique drawn?

In the first phase, shortly after the mothers had signed the consent form to participate in the study, the interviewer investigated their understanding of physical restraint, sedation, and general anesthesia (Table 2).

Immediately after the first phase, the interviewer began the randomization process. It was a block randomization in which the mother had to pull an opaque sealed envelope out of a pile of envelopes, open it, and review the insert to ascertain the child’s treatment assignment. There were 48 envelopes that were divided equally among the three techniques. After the contents of some of the envelopes that consisted of the names of each of the techniques were revealed, they were shuffled in front of the mothers to ensure research transparency; the mothers were not able to watch the envelopes because the sequence of shuffling motions was continuously changed. Each mother had an equal chance of drawing any of the techniques since there were an equal number of envelopes for each treatment group.

Each mother selected one envelope and discovered the intervention group assigned along with the interviewer, who then conducted the second phase of the interview to record the feelings experienced by each mother following the drawing (Table 2). Two mothers who did not accept the randomization result excluded their child from the RCT, but they agreed to allow their interviews to be used in the qualitative analysis. Children who were excluded from the RCT sample received proper dental care at NESO.

All interviews were audio recorded in mp3 files and later transcribed verbatim by the same interviewer.

### Records analysis

The interviewer and another researcher trained in qualitative methods separately analyzed the transcripts using a conventional qualitative content analysis [15]. After an exhaustive reading of each transcript, they independently developed codes based on text excerpts that described the mothers’ feelings about the informed consent and randomization processes. Then, in a consensus meeting, they attempted to reach a satisfactory agreement regarding the codes; any differences that were encountered were resolved through discussion. Thereafter, the codes were organized into empirical subcategories. These subcategories were then refined into categories, and subsequently, themes were developed. To validate the analysis, the results were further discussed at several seminars and conferences with other professionals experienced in qualitative research and/or in the topic of the study. Transcripts were returned to the mothers when there were any questions about their answers.

## Results

The respondents were 15 mothers who were 20 to 45 years old and had levels of formal education ranging from incomplete elementary school to complete high school. Their children, eight boys and seven girls, were between 17 and 36 months of age.

The results of the randomization are displayed in Table 3. After the second phase of the interview, two mothers were excluded from the trial because they did not accept the group to which their children had been assigned, but they were not excluded from this qualitative study. The interviews lasted from 15 to 30 minutes.

**Table 3:** Mothers’ expectations and results of randomization involving the three advanced behavior guidance techniques

**Mother ID**	**Mother’s expectation**	**Group assigned**
M1	General anesthesia	Physical restraint
M2	General anesthesia	General anesthesia
M3	Sedation	Sedation
M4	Sedation	Physical restraint
M5	Sedation	Sedation
M6	General anesthesia	Physical restraint
M7	Sedation	Sedation
M8	Sedation	Physical restraint
M9	General anesthesia	Physical restraint
M10	General anesthesia	General anesthesia
M11	General anesthesia	Sedation
M12	Physical restraint	General anesthesia
M13	General anesthesia	Physical restraint
M14	Unsure	Physical restraint
M15	General anesthesia	General anesthesia

One major theme emerged during the analysis: Understanding, attitudes and feelings regarding the consent to and randomization of advanced behavior guidance techniques. This theme was comprised of three main categories that were extracted from the mothers’ responses: Understanding of advanced behavior guidance techniques, vulnerability to the randomization process, and mothers’ feelings before and after the drawing. These main categories were built on the identified subcategories and categories (Table 4) and are explained in the following subheadings; quotations from the mothers’ responses illustrate the subcategories. Each mother was identified by the letter “M” plus a number to maintain anonymity.

**Table 4:** Content analysis outcomes illustrating mothers’ perceptions of the informed consent and randomization processes

**Theme**	**Main categories**	**Categories**	**Subcategories**
Understanding of and attitudes and feelings towards consent to undergo treatment and randomization of advanced behavior guidance techniques*	Understanding of advanced behavior guidance techniques	Little understanding	Confusion in defining techniques
		Misunderstanding	Questions after signing the consent form
			Definition unknown
	Vulnerability to the randomization process	Before the drawing	Acceptance
			Questioning
		During the drawing	Sharing responsibility with child
	Mothers’ feelings before and after the drawing	Before the drawing	Faith in God
			Fear
			Powerlessness to choose
		After the drawing	Faith in God
			Fear
			Powerlessness to choose
			Relief from/increase in pressure

*Advanced behavior guidance techniques = physical restraint, sedation and general anesthesia.

### Understanding of advanced behavior guidance techniques

This main category represents how well mothers understood the explanations of the advanced behavior guidance techniques (physical restraint, sedation, and general anesthesia) that they had been given during the informed consent process. It is represented by ‘a little understanding’ and ‘misunderstanding’.

The ‘a little understanding’ category included mothers who could give some information about the techniques, but not in as much depth as the explanation they received during the informed consent process. Of the 15 mothers interviewed, eight demonstrated a marginal understanding of the techniques to which their children could be assigned:

I understood sedation. A medicine is given, he sleeps . . . there are children that sleep and others do not. (M1)

Physical restraint wraps the child in a sheet to prevent injury. (M7)

I understood that general anesthesia could do everything at once. (M15)

Seven mothers had difficulty understanding the explanations of the behavior guidance techniques and asked for more information after they had already signed the consent form: “I do not know, and what does that mean? Does the child sleep in general anesthesia? I think it would be better. I do not know anything”. (M9)

Other mothers did not understand any of the information and therefore could not explain the techniques: “She [dentist] kind of explained it to me. No, she explained it well. She talked about the anesthesia – giving syrup that makes you sleepy. Or general anesthesia, when the child will go to the doctor” (M11).

### Vulnerability to the randomization process

This category refers to mothers’ attitudes toward the RCT drawing. The result, by lot, is unpredictable, so this theme relates to mothers’ reactions against the inevitable results of randomization.

Most mothers accepted the random assignment, but two questioned it.

So in this case there is no choice? Is it by lot? (M11)

Just one question: Why can’t we choose? (M12)

Mother M12 reported that her daughter would not receive the treatment if the technique selected was not the one she desired, as demonstrated by the statement below:

. . . Oh, I would not want [to do it]. I came here with my heart in my hand thinking about it. I thought I could choose to come here and say ‘I do not want general anesthesia.’ The doctor at the health center had already said that a sedative might be necessary, so I was already thinking ‘I will not allow it.’ (M12)

Mother M11 transferred the responsibility for drawing the envelope to her toddler, saying: “[Son,] take one, which one do you want? Just take one, stick your finger in here and take it”. (M11)

### Mothers’ feelings before and after the drawing

This main category was divided into two separate categories: pre- and post-drawing responses.

There were three subcategories that comprised the mothers’ pre-drawing responses: faith in God, fear, and powerlessness to choose.

Faithful mothers had absolute confidence that God would help them and indirectly choose the best envelope for their children: “Whatever God prepares for him is fine with me . . . I have talked to God because He knows what is best for him, doesn’t He?” (M4)

Fearful mothers were afraid that the insert in the envelope would determine a group that they would not choose; they felt psychologically threatened:

At the beginning I was scared, we just get scared, I was scared. (M1)

Oh, fear of what I want not happening. (M10)

Despite their fears and beliefs, powerless mothers felt they had to accept the intervention because their children needed the dental treatment:

My goal is for [treatment] to be done because he will lose his teeth, and a child this age without teeth . . . My choice is for him to be treated. (M11)

It cannot continue the way it is. I am like really afraid of general anesthesia, but, as others say, if it is necessary, what can I do? . . . As I am receiving the treatment for free I accept what we get. And I have no choice. (M3)

Following the drawing, mothers were asked if their expectations had been met. In six cases, the techniques they had drawn corresponded to the dental treatment they would have chosen for their children (Table 3). Mothers’ responses were organized into subcategories representing feelings that replicated those experienced before the drawing and the following new codes were identified: faith in God, fear, powerlessness to choose, and relief from or an increase in pressure. The mothers’ faith was related to a feeling that God had heard their wishes: “Fine… [Gave her daughter a kiss]… [Silence and crying]… We must have great faith and never lose hope.” (M3)

At that point, the fears that were expressed were associated with a concern that something could go wrong: “Now I'm worried how it will be that day.” (M2); “. . . is there a risk of death?” (M15). Again, feelings of powerlessness appeared that were related to the mothers’ desire to resolve their children’s oral problems:

It will be difficult for me, I know it is not easy for me to hold him, he will cry a lot, I already know how he is, but it is okay . . . I imagine it will be very difficult but I totally need him to have this treatment because he has so much tooth decay. (M4)

Mothers also externalized the pressures that they were feeling. Some of them were relieved after the drawing because their child had been assigned the treatment they had wanted:

Relieved. I feel like jumping for joy. That is great. [kisses the child]. [laughs]. (M5)

We feel relief, knowing he will not feel the treatment so much. (M11)

Other mothers felt increased pressure. They felt dissatisfied because something that they had expected had not materialized:

We would like to have gotten the general anesthesia . . . Now we have to do it with the sheet [passive restraint]. (M9)

Oh, no! (M12)

M13 cried, but did not say anything after the drawing.

## Discussion

This qualitative study suggests that the informed consent and randomization processes should be better designed in clinical trials that involve therapeutic comparisons and emotional issues. In this study, mothers felt excessive pressure as a result of the informed consent procedures, the randomization method, and their children’s dental problem.

Some mothers showed that they understood the explanations provided during the informed consent process, but others revealed that they did not understand them. In this trial, the dentist provided face-to-face oral explanations associated with the consent form, which usually optimizes understanding of the written information [12,16,17]. However, nearly half of the mothers interviewed could not describe what they had understood from the informed consent process; this level of confusion also was observed in another study [12]. It can be hypothesized that the mothers were already stressed before they were invited to participate in the trial because they were seeking dental treatment to resolve their children’s oral problems.

Another factor that may have contributed to the misunderstanding of the information contained in the consent form was the concern generated by the risks associated with the advanced behavior management techniques. Thus, the obtainment of dental treatment was something that produced great expectation and anxiety in the mothers. It is well established that high emotions hinder the assimilation of new and/or complex information that is contained in a consent form [16,18].

The present results are supported by the findings of other studies. One study [19], which investigated whether parents of children who had undergone general anesthesia in a hospital had fully understood the proposed treatment, revealed that 40% of the information contained in the informed consent was not comprehended by the parents. While the parents’ understanding seemed to improve only on the day of treatment, 19% did not understand exactly what would happen to their children before the treatment was administered. These findings are consistent with the present results, in which some mothers, after signing the consent form, were unable to cite or explain any of the techniques that had been proposed by the team to guide the child’s behavior during dental care. One solution for this issue has been proposed: the simplification of consent forms and the placement of additional emphasis on verbal explanations of the study procedures [12,13].

Reports addressing the feelings of participants about the randomization process are scarce, but researchers should not disregard this topic. Although most of the mothers in this study did not question the reason for using a drawing technique, they felt pressure to determine what would occur next. Feelings of faith, fear, and powerlessness prior to and following the drawing were observed in the mothers. Their faith was related to fate, luck and confidence, feelings that were also identified in another study [5] of men who had been randomized into three groups for the treatment of symptoms of benign prostatic disease. In fact, religious belief helps families to attribute meaning to their experiences. This result is supported by another qualitative study [20], which found that families of children with a life-threatening disease perceived God or a higher being as having healing power.

In this study, early childhood caries disturbed families to such an extent that some mothers felt powerlessness to decide which behavior management guidance they would prefer; they forgot their fears and their only desire was to have their children relieved from their toothache and its consequences on family dynamics. The willingness of most mothers to accept the technique they were allocated was perhaps directly related to the urgency of their need for dental treatment for the child or the fear that the opportunity to be treated at a public institution with specialized dental care would be lost. For these mothers, the most important thing was to resolve their children’s dental problems. Similar results were found in a study [21] of parents of children with cancer, in which parents were found to be concerned with the effectiveness of the treatment itself, and not with research protocols and possible randomization techniques. Interestingly, parents in other studies [13,22] also were determined to have signed the consent form because they trusted the medical team although the main factor that motivated parents to allow their children to participate in a clinical trial was the “direct benefits for their children” [23].

Nevertheless, another group of mothers feared one or another of the advanced behavior guidance techniques. The fear of anesthesia has been recognized as a barrier to surgical care in low- and middle-income populations [24], although physical restraint also may be poorly accepted by parents [6]. None of the mothers in our study seemed to understand that randomization was necessary because the researchers did not know which intervention would be the most effective for their children’s treatment – physical restraint, moderate sedation, or general anesthesia. When the results of the randomization did not correspond to the mothers’ expectations, with the exception of two cases, they looked disappointed but accepted the assignment. Another study investigated recruitment processes across a spectrum of trials of medicines for children; it reported that parents valued safety above other benefits to their children and declined the randomized intervention if they felt a passing sense of discomfort [12].

Throughout the data collection and analysis, efforts were made to warrant the credibility of this study. To avoid the inhibition of the respondents, a researcher with a background in psychology who did not participate in the informed consent process conducted the interviews. During the course of the analysis, the transcripts were studied exhaustively and returned to the mothers if any questions arose. The interpretations of the findings were scrutinized through discussions between the two authors as well as with other professionals familiar with the methodology and the study topic. However, the interviews were conducted in the dental clinic, which may have evoked a sense of insecurity in the respondents. Also, the young child occasionally accompanied the mother during the interview, requiring the mother to divide her attention between the researcher and her child. Another possible methodological limitation is that we interviewed low-income mothers with limited formal education, a characteristic that makes them more vulnerable. The results may have been different if mothers from different socioeconomic backgrounds had been included, and thus, there should be further study addressing this issue.

All in all, this study indicates that informed consent and randomization are crucial steps in a trial from the participants’ point of view and should be more thoroughly discussed by investigators who conduct RCTs. Researchers should invest more time to create resources that better explain consent and randomization to participants in RCTs, particularly those involving therapeutic comparisons and emotional issues.

## Conclusions

Mothers’ perceptions of the informed consent and randomization processes in a clinical trial were characterized by:

1. Misunderstandings of these research steps, vulnerability in the acceptance of the intervention drawn, and contradictory feelings of faith, fear, powerlessness and pressure.

2. A predisposition to overlook their feelings to ensure the completion of their children’s dental treatment.

## Abbreviations

RCT: Randomized clinical trial; NCT: Number in clinicaltrials.gov; IRB: Institutional review board; NESO: Dental sedation center; UFG: Federal University of Goias.

## Competing interests

The authors declare that they have no competing interests.

## Author’s contributions

AAC and LRC made substantial contributions to the conception and design of the trial, drafted the manuscript and revised the manuscript critically for intellectual content. All authors read and approved the final version.

## Pre-publication history

The pre-publication history for this paper can be accessed here:

http://www.biomedcentral.com/1472-6939/14/52/prepub
